# Blocking Nonspecific Interactions Using Y-Shape Poly(ethylene glycol)

**DOI:** 10.3390/ijms241512414

**Published:** 2023-08-04

**Authors:** Zhengyu Xu, Qingtai Li, Yaying Huang, Kaiqiang Guo, Bin Xue, Yi Cao, Yiran Li

**Affiliations:** 1 Collaborative Innovation Center of Advanced Microstructures, National Laboratory of Solid State Microstructure, Department of Physics, Nanjing University, Nanjing 210093, China; dz1922031@smail.nju.edu.cn (Z.X.); lqt861563391@163.com (Q.L.); mg21220199@smail.nju.edu.cn (Y.H.); 191840079@smail.nju.edu.cn (K.G.); xuebinnju@nju.edu.cn (B.X.); 2 Jinan Microecological Biomedicine Shandong Laboratory, Jinan 250021, China; 3 Medical School, Nanjing University, Nanjing 210093, China

**Keywords:** single-molecule force spectroscopy, atomic force microscopy, nonspecific interaction, surface modification

## Abstract

Nonspecific interactions play a significant role in physiological activities, surface chemical modification, and artificial adhesives. However, nonspecificity sometimes causes sticky problems, including surface fouling, decreased target specificity, and artifacts in single-molecule measurements. Adjusting the liquid pH, using protein-blocking additives, adding nonionic surfactants, or increasing the salt concentration are common methods to minimize nonspecific binding to achieve high-quality data. Here, we report that grafting heteromorphic polyethylene glycol (Y-shape PEG) with two inert terminates could noticeably decrease nonspecific binding. As a proof-of-concept, we performed single-molecule force spectroscopy and fluorescence staining imaging experiments to verify the feasibility of Y-shape PEG in blocking nonspecific interactions. Our results indicate that Y-shape PEG could serve as a prominent and efficient candidate to minimize nonspecificity for scientific and biomedical applications.

## 1. Introduction

Preventing nonspecific interactions or adsorption is a ubiquitous and persistent challenge in various fields, such as molecular biology, cellular biology, bioengineering, and biomedicine [1,2,3,4,5,6,7]. For instance, in the experimental study of molecular interactions, the prevention of nonspecific adsorption can ensure that the specific binding interactions to the molecule of interest are precisely targeted and investigated [8]. Nonspecific binding introduces artifacts and uncertainty in data acquisition, which hinders the collection of meaningful data. In addition, blocking nonspecific binding is one of the most critical steps before immunostaining, Western blot, and ELISA experiments, which can significantly reduce background staining and improve the signal-to-noise ratio [9,10]. Moreover, the interactions of impurity molecules lead to decreased sensitivity and selectivity in the case of biosensors [6,11,12]. For implanted medical materials [13,14,15], nonspecific interactions may lead to blood clotting [16], inflammation reactions, or corrosion [17] due to the interactions of different enzymes [18,19].

In particular, it is especially crucial to avoid nonspecific interactions in single-molecule measurements, including optical tweezers [1,20,21], magnetic tweezers [21,22], atomic force microscopy (AFM) [21,23,24], and microneedle manipulation [25]. These techniques rely on specific molecular recognition to study the dynamics and conformational changes of biomacromolecules in physiological environments [26,27,28,29]. On the one hand, the ranges of common nonspecific interactions can be several hundred nanometers in length and nanonewtons in force, which occludes the targeted signal. On the other hand, nonspecifically adsorbed impurities can easily occupy specific binding sites, making it difficult to observe the desired dynamics and conformational changes of the molecules. Some methods have been developed to reduce nonspecific interactions, including biomolecule blocking [14,30,31,32,33,34,35], hydrophilic cleaning [36], natural polymer surface modification [37], and organic polymer surface modification [38,39,40,41,42,43,44]. BSA usually shields nonspecific adsorption of impurities or contaminants on both hydrophobic and hydrophilic surfaces. However, most BSA contains globulins or endotoxins, which may bind nonspecifically to impurity molecules [30,31]. Casein is also used to block nonspecific interactions in tissue immunofluorescence because of its good blocking performance and economical cost [34]. In addition to using inert proteins, grafting hydrophilic polymers on the surface of microfluidic chips, sensor chips, immune probes, and other macroscopic objects is also a commonly used method for reducing nonspecific adsorption of microorganisms and biomolecules, including polyethylene glycol (PEG), chitosan, dextran, poly(oxyethylene), poly(ethyleneimine), poly(acrylamide), poly(glycerol), zwitterionic polymers, poly(vinylpyrrolidone), etc. A longer polymer chain could cover more surface area, thus giving rise to better protection. However, if the polymer chain is too long or the modification density is too high, it will cause intermolecular or intramolecular entanglement [36,37,38,40,43]. In addition, ethanolamine and cysteine can be used as blocking agents in covalently bound protein coupling procedures, but it is possible to negatively affect the antigen [44]. Thus, issues such as general shielding effects, insufficient maintenance time, and uncontrollable shielding locations remain challenges [45].

Here, we present a method that uses Y-shape polyethylene glycol (Y-mPEG) to block nonspecific interactions. We used single-molecule force spectroscopy (SMFS) and fluorescence staining experiments in which shielding nonspecific interactions is necessary and a critical step to improve data quality. By shielding the nonspecific binding of substrates and cantilevers using Y-mPEG, the nonspecific interaction peaks were much weaker, efficiently clarifying the specific binding. This led to an increase in the sample rate of single-molecule events, which was even higher than that using linear-shaped PEG. In addition, we further demonstrated that blocking nonspecific interactions in fluorescence staining of cells could minimize the aggregation of fluorescent dyes in the background but had little side effect on living cells because of the biocompatibility and controllable modification of PEG. We anticipate that nonspecific interaction blocking based on Y-mPEG can be widely applied to various scenarios, including but not limited to single-molecule experiments, biosensors, immunological assays, and medical materials.

## 2. Results and Discussion

### 2.1. Design of Blocking Nonspecific Interactions Using Y-mPEG in SMFS

**Figure 1:**
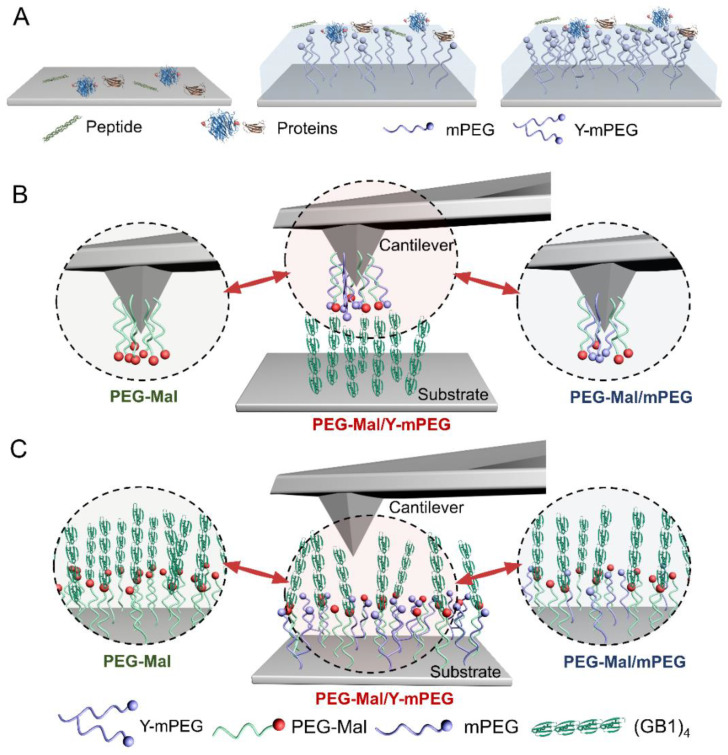
Design of blocking nonspecific interactions using Y-shape PEG. (**A**) Schematic of blocking nonspecific interactions using Y-shape PEG. (**B**,**C**) Schematic of blocking nonspecific interactions in SMFS using Y-shape PEG (PEG-Mal/Y-mPEG) by modifying the cantilever (**B**) or substrate (**C**). The cantilevers or substrates without blocking (PEG-Mal) or with blocking using linear PEG (PEG-Mal/mPEG) were used as controls.

Typically, substances such as peptides, proteins [46], and other hydrophobic molecules aggregate and absorb on substrates or implanted medical pipelines via nonspecific interactions, including but not limited to electrostatic, hydrophobic, Van Der Waals, and steric interactions in aqueous environments (left of Figure 1A). The binding of nontarget molecules may hinder the reliability of experimental results. Hence, inert hydrophilic polymers, such as PEG, have been used to block nonspecific interactions by coating substrates. The grafted inert PEG layer can reduce the exposure possibility of active substrates to nontarget molecules in solution and generate a hydration layer on the substrates, consequently preventing the absorption of different molecules through nonspecific interactions [47,48]. Increasing the density of PEG on the substrates can enhance the prevention of nonspecific absorption. As illustrated in the middle and right of Figure 1A, the cover area of hydrophilic chains would increase when modifying the substrates using Y-shape PEG instead of linear PEG at the same molecular density since the grafted PEG has more free branch chains (Figure S1).

Based on this concept, Y-mPEG is the best candidate to effectively occupy the free space of cantilevers or substrates and block nonspecific interactions in SMFS experiments. Typically, in single-molecule tests, target molecules can be modified on either the AFM cantilever or substrate surface. To this end, we designed experiments utilizing Y-mPEG for surface modification to shield nonspecific interactions in SMFS (Figure 1B,C) and used different modification strategies to verify the effect of blocking. For the blocking of nonspecific binding on cantilevers, the amino-modified cantilever (middle of Figure 1A) is connected with NHS-PEG-maleimide (denoted PEG-Mal) and Y-shape PEG-SC (denoted Y-mPEG, Figure S2) via amine-active ester reactions. The engineered protein composed of four repeated GB1 domains with a C-terminus of L-cysteine (denoted as (GB1)_4_-Cys) is absorbed on the substrates. When the cantilever extends to the substrate surface, (GB1)_4_-Cys can covalently connect to PEG-Mal via maleimide–thiol conjugation [49]. During the retraction of the cantilever, (GB1)_4_-Cys unfolded under stretching, and the force–distance curves were recorded. The introduction of Y-mPEG can decrease the density of PEG-Mal on the cantilever and provide a shielding layer to minimize nonspecific adsorption, thus decreasing the pickup rates of stretching multiple proteins simultaneously. To block nonspecific binding on substrates, the substrates were modified with PEG-Mal and Y-mPEG (Figure 1C). (GB1)_4_-Cys was added to the substrate and linked with PEG-Mal via maleimide–thiol conjugation. After removing the unbound protein, the cantilever extends to the substrate surface and picks up the protein. Similarly, the pickup rates of multiple protein absorption and the number and strength of events brought by nonspecific binding were supposed to decrease due to the blocking of Y-mPEG. The cantilevers and substrates modified using PEG-Mal or PEG-Mal/NHS-linear PEG (mPEG) were used as controls (left and right of Figure 1B,C).

### 2.2. Surface Modification Using Y-mPEG

**Figure 2:**
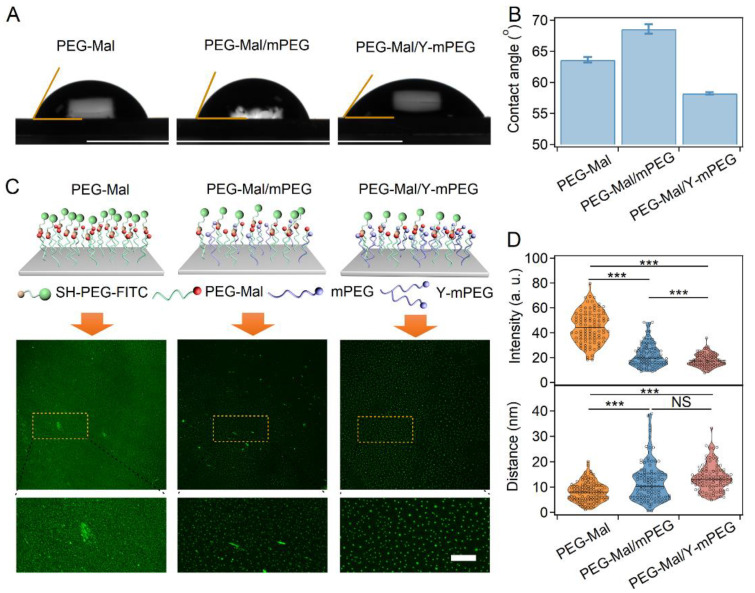
Characterization of the Y-mPEG modification on silica substrates. (**A**,**B**) Images (**A**) and average values (**B**) of the static contact angle (SCA) of 16 µL droplets of water on silica substrates modified with different PEGs in air. (**C**) Schematic and fluorescence microscopic images of substrates modified with different PEGs after FITC labeling. Fluorescence spots in the dashed rectangular boxes are shown enlarged. Scale bar: 100 μm. (**D**) Background fluorescence intensities of the images (**top**) and the average distance between the fluorescent spots (**bottom**) in C. Statistical significance was determined by a two-tailed *t*-test. NS: *p* > 0.05; ***: *p* < 0.001.

We first investigated the feasibility of surface modification using Y-mPEG. The SiO_2_ substrates were successfully modified solely with PEG-Mal, PEG-Mal/mPEG, and PEG-Mal/Y-mPEG, as indicated by the peak at approximately 2883 cm^−1^ in the Raman spectrum (Figure S3). As indicated by the evaluation of solid surface static contact angles, we compared the hydrophilicity of substrates with different modifications. The surface contact angle of the PEG-Mal/Y-mPEG substrate was slightly lower than that of the PEG-Mal substrate, while that of the PEG-Mal/mPEG substrate was much higher. Each experiment was repeated three times. The error bars indicate the standard deviation (Figure 2A,B). This indicated that the multibranched-chain structure of Y-mPEG endows it with stronger hydrophilicity when acting as a surface coating but without additional modification steps, which can effectively inhibit nonspecific interactions.

After successfully modifying the substrate surface with different PEGs, we further labeled the substrates with a fluorescent marker (fluorescein isothiocyanate-PEG-thiol, denoted as SH-PEG-FITC). Laser confocal microscopy images of the substrate surface revealed that when solely PEG-maleimide was used for modification, the fluorescent labeling preferred to form continuous plaques or dense dots with a high background fluorescence. In contrast, a uniform distribution of fluorescent spots with low background fluorescence was observed for the substrates modified with PEG-Mal/mPEG and PEG-Mal/Y-mPEG (Figure 2C). This trend was further confirmed by statistical analysis of the background fluorescence intensity and the average distance between the fluorescent spots (Figure 2D). The distribution of the average distance between the fluorescent spots was the highest in the PEG-Mal/Y-mPEG group, suggesting the excellent performance of nonspecific interaction inhibition. These results indicated that surface modification using Y-mPEG can contribute to the uniform distribution of target molecules, which can benefit the following SMFS experiments.

### 2.3. Blocking Nonspecific Interactions by Modifying AFM Cantilevers with Y-mPEG in SMFS

**Figure 3:**
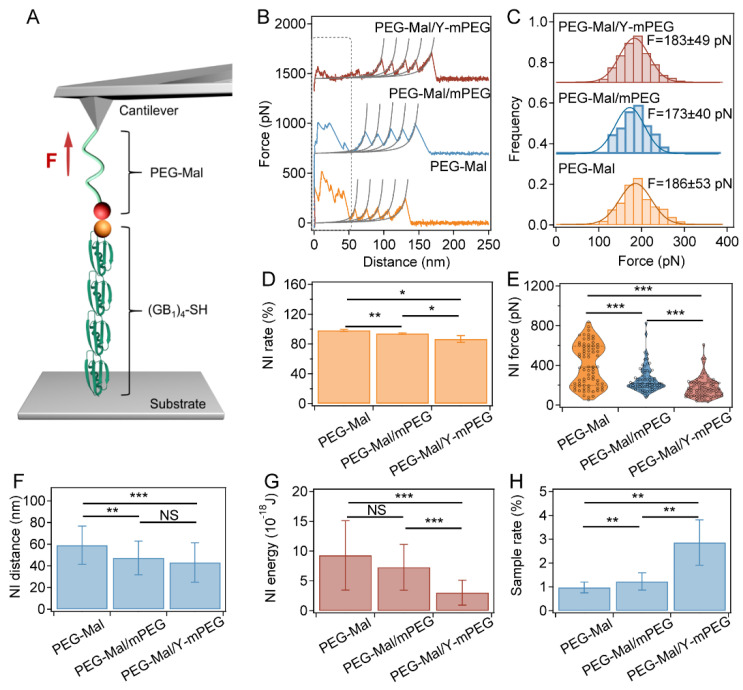
SMFS results for the (GB1)_4_ polyprotein using different PEG modifications on the cantilever to block nonspecific interactions. (**A**) Schematic of the single-molecule force spectroscopy of (GB1)_4_ unfolding under force using the cantilever modified with different PEGs. (GB1)_4_ was anchored to a glass surface and picked up using a PEG-Mal-modified cantilever via thiol–maleimide chemistry. (**B**) Typical retract force–distance curves for the unfolding of (GB1)_4_ using cantilevers modified with different PEGs. Each peak was fitted by the worm-like chain (WLC) model of polymer elasticity. The initial peaks at an extension range of 0–50 nm correspond to nonspecific interactions, and the next four peaks with a Δ*L_c_* of 18–19 nm correspond to the unfolding of GB1 domains (as shown in Figure S7A). (**C**) Unfolding force histograms of (GB1)_4_ using cantilevers modified with different PEGs. The Gaussian fitting shows average rupture forces of 183 ± 49 pN, 173 ± 40 pN, and 186 ± 53 pN, respectively. (**D**) Probabilities for the observation of nonspecific interactions during (GB1)_4_ unfolding using cantilevers modified with different PEGs (*n* > 10,000). (**E**–**G**) Peak force (**E**), fracture length (**F**), and energy (**G**) of the nonspecific interactions calculated from the force–distance curves using cantilevers modified with different PEGs (*n* = 100). The cantilevers prepared without blocking nonspecific interactions using PEG or with blocking using linear PEG (PEG-Mal/mPEG) were used as controls. (**H**) Pickup rates of (GB1)_4_ unfolding curves using cantilevers modified with different PEGs (*n* > 10,000). Statistical significance was determined by a two-tailed *t*-test. NS: *p* > 0.05; *: *p* < 0.05; **: *p* < 0.01; ***: *p* < 0.001.

Next, we investigated the effects of nonspecific interaction inhibition by comparing the nonspecific interaction peak and the specific (GB1)_4_-Cys protein unfolding peak in the SMFS force curves with different PEG-modified cantilevers. We collected more than 10,000 pieces of data in each group, and each experiment was conducted five times. In these experiments, inert Y-mPEG, together with active linear maleimide PEG, were grafted onto the cantilever surfaces. For the SMFS experiment with the modified cantilever (Figure 3A), the four GB1 domains will sequentially unfold during the retraction of the cantilever. The typical retracted force–distance curves containing sawtooth-like peaks are shown in Figure 3B. The unfolding forces of GB1 determined by SMFS using different PEG modification strategies were 183 ± 49 pN, 173 ± 40 pN, and 186 ± 53 pN, respectively (Figure 3C), which is consistent with previous reports [50]. The positions of the first GB1 unfolding peaks determined by SMFS using different PEG modification strategies were approximate (Figure S4A). These results indicate that grafting inert Y-shape or linear PEG has little influence on the target protein native structure or single-molecule measurements. In addition, a nonspecific interaction region was observed at the beginning of retracting force-distance curves from modified cantilevers (PEG-Mal, PEG-Mal/mPEG, and PEG-Mal/Y-mPEG, respectively highlighted by the dashed rectangular box in Figure 3B). However, the nonspecific adhesion range and force strength vary with different PEG-modified cantilevers. For the force curves obtained from cantilevers modified with solely PEG-Mal, there were large nonspecific interaction peaks at the beginning stage, which in many cases even overshadowed the unfolding peaks of the (GB1)_4_ protein. In contrast, after being modified with PEG-Mal/mPEG, the nonspecific interaction peak decreased slightly. When the cantilever was modified with PEG-Mal/Y-mPEG, both the force value and the strength of the nonspecific interaction peak were significantly reduced and well separated from the (GB1)_4_ unfolding signal. The occurrence rate (NI rate), peak value (NI force), width (NI distance), and adhesive energy (NI energy) of nonspecific interactions of the PEG-Mal/Y-mPEG group were remarkably lower than those of the PEG-Mal and PEG-Mal/mPEG groups (as shown in Figure 3D–G and Figure S5). Correspondingly, the sampling rate of single-molecule events for protein unfolding in the PEG-Mal/Y-mPEG group was also greater than those in the PEG-Mal and PEG-Mal/mPEG groups (Figure 3H).

### 2.4. Blocking Nonspecific Interactions by Modifying Substrates with Y-mPEG in SMFS

**Figure 4:**
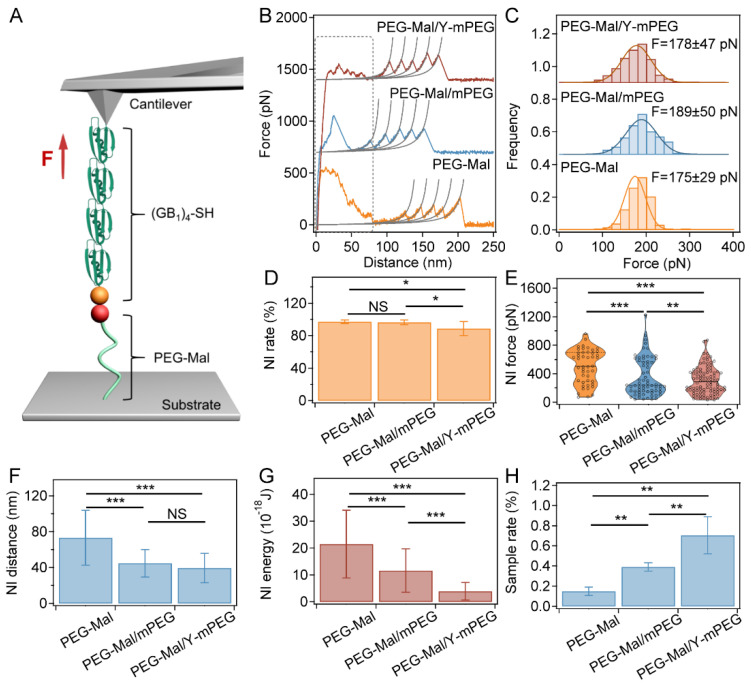
SMFS for the (GB1)_4_ polyprotein by blocking nonspecific interactions using different PEGs on substrates. (**A**) Schematic of the SMFS of (GB1)_4_ unfolding under force on substrates modified with different PEGs. (GB1)_4_ was anchored to substrates modified with different PEGs via thiol-maleimide chemistry and picked up using a silicon nitride cantilever. (**B**) Typical retract force–distance curves for the unfolding of (GB1)_4_ on substrates modified with different PEGs. Each peak was fitted by the worm-like chain (WLC) model of polymer elasticity. The initial peaks at an extension range of 0–65 nm correspond to the nonspecific interactions, and the next four peaks with a Δ*L_c_* of 18–19 nm correspond to the unfolding of GB1 domains (as shown in Figure S7B). (**C**) Unfolding force histograms of (GB1)_4_ on substrates modified with different PEGs. The Gaussian fitting shows average rupture forces of 178 ± 47 pN, 189 ± 50 pN, and 175 ± 29 pN, respectively. (**D**) Probabilities for the observation of nonspecific interactions during (GB1)_4_ unfolding using substrates modified with different PEGs (*n* > 10,000). (**E**–**G**) Peak force (**E**), fracture length (**F**), and energy (**G**) of the nonspecific interactions calculated from the retracted force–distance curves using cantilevers modified with different PEGs (*n* = 100). The substrates prepared without blocking nonspecific interactions using PEG or with blocking using linear PEG (PEG-Mal/mPEG) were used as controls. (**H**) Pickup rates of (GB1)_4_ unfolding curves using substrates modified with different PEGs (*n* > 10,000). Statistical significance was determined by a two-tailed *t*-test. NS: *p* > 0.05; *: *p* < 0.05; **: *p* < 0.01; ***: *p* < 0.001.

For the SMFS experiment using substrates modified with different PEGs (Figure 4A), similar trends were found. We collected more than 10,000 pieces of data in each group, and each experiment was conducted five times. By modifying the substrates with PEG-Mal/Y-mPEG, the nonspecific interaction peak significantly decreased (Figure 4B). The lowest nonspecific interaction occurrence rate (NI rate), peak value (NI force), width (NI distance), and adhesive energy (NI energy) were also observed in the PEG-Mal/Y-mPEG group (Figure 4D–G and Figure S6), with the statistical unfolding force remaining consistent (Figure 4C). The positions of the first GB1 unfolding peaks determined by SMFS using different PEG modification strategies were approximate (Figure S4B). The sampling rate of single-molecule events in the PEG-Mal/Y-mPEG group was also greater than that of mixed modification of PEG-maleimide with linear PEG and PEG-maleimide modification alone (Figure 4H). Our SMFS results clearly showed that the Y-mPEG could greatly improve SMFS data quality.

### 2.5. Blocking Nonspecific Interactions Using Y-mPEG in Fluorescence Staining

**Figure 5:**
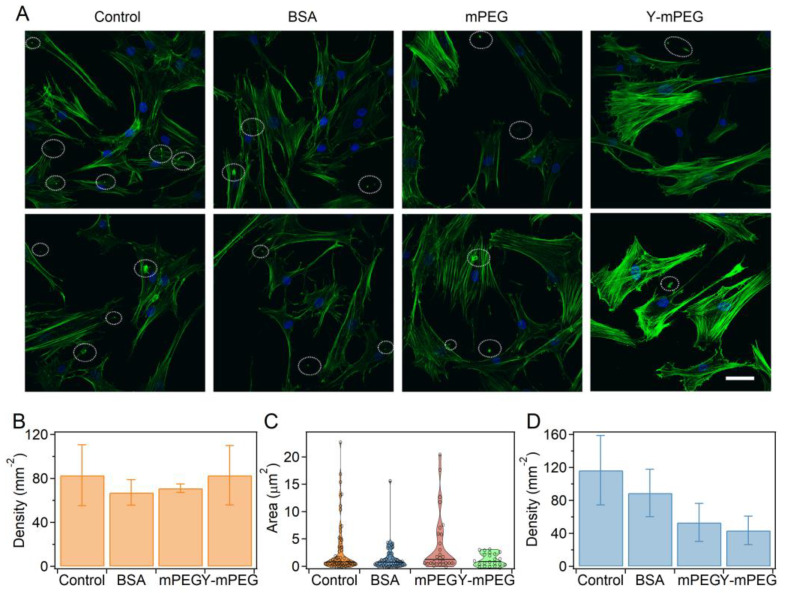
Blocking nonspecific interactions using Y-mPEG in the immunofluorescence staining of hMSCs. (**A**) Fluorescent images of hMSCs by blocking nonspecific interactions using different PEGs during staining. The cytoskeleton and nucleus of cells were stained with phalloidin (green) and DAPI (blue) after 24 h of culture. The scale bar is 50 µm. The fluorescence spots caused by aggregation of fluorochrome due to nonspecific binding are highlighted with dashed boxes. Staining without blocking nonspecific interactions was used as a control. (**B**) Densities of cells in different groups. (**C**,**D**) Area (**C**) and densities (**D**) for the fluorescence spots of nonspecific binding in different groups.

Blocking nonspecific binding is also important in cellular biology. Here, we demonstrated the application of blocking nonspecific binding using Y-mPEG with fluorescence staining as an example. Typically, human mesenchymal stem cells (hMSCs) cultured on cell culture plates were co-incubated with different PEGs for 45 min to block nonspecific binding between substrates/cells and fluorochromes. Then, the cell nucleus and cytoskeleton of hMSCs were stained with 4′,6-diamidino-2-phenylindole (DAPI) and phalloidin, respectively. The cells stained without nonspecific interaction blocking or with blocking using BSA were prepared as controls. We used BSA here because it is one of the most widely used blocking reagents in biological specimen staining [30]. As shown in Figure 5A,B, the density and cellular morphology of hMSCs in different groups remained consistent, indicating that blocking using different molecules did not lead to the exfoliation of cells. The fluorescence intensity of the cells in the immunofluorescence experiment of the Y-mPEG group did not decrease, indicating that the specific binding of the fluorescent dye was not affected. However, obvious fluorescence spots at the microscale (highlighted with dashed boxes) were observed on the background and cytoskeleton in the immunofluorescent images. We anticipate that these fluorescence spots can be attributed to the aggregation of fluorochromes due to residual nonspecific binding. The diameter and density of the nonspecific binding spots in the BSA, mPEG, and Y-mPEG groups were much lower than those in the uncoated group (Figure 5C,D). More importantly, the Y-mPEG group exhibited the lowest diameter and density of fluorescence spots according to the statistical analysis, indicating the best effect of blocking nonspecific interactions using Y-mPEG. All these results demonstrated the advantages of Y-mPEG in blocking nonspecific interactions.

## 3. Materials and Methods

### 3.1. Materials

Y-shape PEG-SC (Y1PT02) was purchased from SinoPEG, Inc. (cat: 06020502054, Xiamen, China). Maleimide-PEG-NHS was purchased from Nanocs, Inc. (cat: PG2-MLNS-5k-2, USA). Methoxy-PEG-succinimidyl glutarate (M-PEG-SG) was purchased from JenKem Technology (cat: M-SG-5000, Beijing, China). SH-PEG-FTIC was purchased from ToYongBio (cat: 06020502054, Shanghai, China). The hMSC line was kindly provided by the Cell Bank of the Chinese Academy of Sciences (Chinese Academy of Sciences, Shanghai, China). The cell culture medium α-MEM (cat: 310-010-CL) was purchased from Wisent, Nanjing, China. Bovine serum albumin (BSA, cat: ST2254) was purchased from Beyotime (China), and Triton X-100 (cat: 0694) was purchased from Amresco (Solon, OH, USA). 4′,6-Diamidino-2-phenylindole (DAPI, cat: MBD0015) was purchased from Sigma-Aldrich. Alexa Fluor^TM^ 488 phalloidin (cat: A12379) was purchased from Invitrogen™, Waltham, MA, USA. All other reagents, unless otherwise stated, were purchased from Shanghai Aladdin Biochemical Technology Co., Ltd. (Shanghai, China). All reagents were used without further purification.

### 3.2. Static Contact Angle

Contact angle values were acquired with an optical contact angle meter (JY-82C, Chengde Dingsheng Testing Machine Testing Equipment Co., Ltd., Chengde, China) at room temperature (≈25 °C). A droplet of Milli-Q water (16 µL) in the air was dropped on the substrate with an automatic dispensing controller, and the contact angles were determined automatically using the Young–Laplace fitting algorithm. The average contact angle values were obtained by measuring the sample at five different positions on the substrate. The SiO_2_ substrates were cut into slides (1 × 1 cm^2^) and soaked in freshly prepared chromic acid overnight. After being washed with deionized (DI) water, ethanol, and acetone successively, the substrates were dried under a stream of nitrogen to produce surfaces with exposed hydroxyl groups. These substrates were immersed in an anhydrous toluene solution containing 5% (*v*/*v*) (3-aminopropyl)triethoxysilane (APTES) (Merck, Kenilworth, NJ, USA) at room temperature (R.T.) for 1 h. Then, they were washed with toluene and ethanol, dried under a nitrogen flow, and incubated at 80 °C for 30 min. These substrates were immersed in DMSO containing 0.2 mM NHS-PEG-Mal, 0.2 mM NHS-linear PEG (mPEG), and 0.2 mM Y-shape PEG-SC (Y-mPEG) separately. Finally, these substrates were dried under a stream of nitrogen and stored dry.

### 3.3. Fluorescent Substrate Preparation

Typically, the glass substrates were cut into slides (1 × 1 cm^2^) and soaked in freshly prepared chromic acid overnight. After being washed with deionized (DI) water, ethanol, and acetone successively, the substrates were dried under a stream of nitrogen to produce surfaces with exposed hydroxyl groups. These substrates were immersed in an anhydrous toluene solution containing 5% (*v*/*v*) APTES (Merck, USA) at room temperature (R.T.) for 1 h. Then, they were washed with toluene and ethanol, dried under a nitrogen flow, and incubated at 80 °C for 30 min. These glass substrates were immersed in DMSO containing 0.2 mM NHS-PEG-Mal, 0.1 mM NHS-PEG-Mal, 0.1 mM NHS-linear PEG (mPEG), 0.1 mM NHS-PEG-Mal, and 0.1 mM Y-shape PEG-SC (Y-mPEG), separately. Finally, substrates were immersed in DMSO containing 0.2 mM SH-PEG-FITC for 60 min and imaged using a laser confocal fluorescence microscope (Olympus FV3000, Tokyo, Japan) after drying under a stream of nitrogen.

### 3.4. Preparations of Cantilevers and Substrates in SMFS

For the preparation of cantilevers and substrates used in SMFS by blocking nonspecific interactions using Y-mPEG-modified cantilevers, silicon nitride (Si_3_N_4_) cantilevers (MLCT-D, Billerica, MA, Bruker) were first cleaned with Milli-Q water and then placed in a chromic mixture (chromic acid) at 80 °C for 30 min. After that, the cantilevers were washed with DI water and then ethanol and dried under a stream of nitrogen. Then, the hydroxylated cantilevers were immersed in an anhydrous toluene solution containing 1% (*v*/*v*) APTES for 1 h. After that, they were rinsed with toluene, then ethanol, dried under a nitrogen stream, and incubated at 80 °C for 30 min. Then, these cantilevers were immersed in DMSO containing 0.2 mM NHS-PEG-Mal, 0.1 mM NHS-PEG-Mal, 0.1 mM NHS-linear PEG (mPEG), 0.1 mM NHS-PEG-Mal, and 0.1 mM Y-shape PEG-SC (Y-mPEG), separately. Finally, cantilevers were immersed in PBS (10 mM, pH = 7.4) containing 0.3 wt% (GB1)_4_ and washed with PBS (10 mM, pH = 7.4) three times before the SMFS experiment. For the preparation of substrates, glass substrates were cut into 1 × 1 cm^2^ slides, soaked in a freshly prepared chromic acid overnight, thoroughly washed with deionized (DI) water and ethanol successively, and then dried under a stream of nitrogen before the SMFS experiments.

For the preparation of cantilevers and substrates used in SMFS by blocking nonspecific interactions using Y-mPEG-modified substrates, glass substrates were cut into 1 × 1 cm^2^ slides, soaked in a freshly prepared chromic acid overnight, thoroughly washed with deionized (DI) water, ethanol, and acetone successively, and then dried under a stream of nitrogen to produce surfaces with exposed hydroxyl groups. These substrates were immersed in an anhydrous toluene solution containing 5% (*v*/*v*) APTES (Merck, USA) at room temperature (R.T.) for 1 h. Then, the cantilevers were washed with toluene and ethanol, dried under a nitrogen flow, and incubated at 80 °C for 30 min. Then, these glass substrates were immersed in DMSO containing 0.2 mM NHS-PEG-Mal, 0.1 mM NHS-PEG-Mal, 0.1 mM NHS-linear PEG (mPEG), 0.1 mM NHS-PEG-Mal, and 0.1 mM Y-shape PEG-SC (Y-mPEG), separately. Finally, substrates were immersed in PBS containing 0.3 wt% (GB1)_4_ and washed with PBS (10 mM, pH = 7.4) three times before the SMFS experiment. For the preparation of cantilevers, silicon nitride (Si_3_N_4_) cantilevers (MLCT-D, Bruker) were first cleaned with Milli-Q water and then placed in a chromic mixture (chromic acid) at 80 °C for 30 min. After that, the cantilevers were washed with DI water and ethanol, and dried under a stream of nitrogen before the SMFS experiments.

### 3.5. AFM-Based Single-Molecule Force Spectroscopy (SMFS)

The force spectroscopy experiments were carried out using a commercial JPK ForceRobot 300 AFM system (JPK, Berlin, Germany). Experiments were conducted at R.T. (22 °C) and performed in a 10 mM PBS buffer. Soft silicon nitride MLCT-D cantilevers with a typical spring constant of 30–45 pN nm^−1^ were used for all experiments and calibrated using the thermal tuning method using the data acquisition software from JPK after allowing the cantilever to equilibrate in solution for at least 30 min. To transform the electrical signals from the photodiode to the actual displacement, the optical lever sensitivity and its inverse were determined by pushing the cantilever tip to the glass substrate and assuming that the bending of the cantilever was equal to the movement of the piezoelectric positioner. In a typical pulling experiment, cantilevers were briefly and gently (~300 pN) brought in contact with the functionalized surface, held at the surface for 1 s, and then retracted at a constant velocity of 1 μm s^−1^. The sampling frequency was 10 kHz, and the records were smoothed by the moving average of 10 points. The force–extension curves were recorded using JPK data processing software and were further analyzed by a custom-written procedure in Igor 6.37 (Wavemetric Inc., Portland, OR, USA). We only used the curves containing four peaks of contour length increments of ~18 nm corresponding to the unfolding of the GB1 domains for data analysis. The contour length increment was determined by fitting the force peaks using the worm-like chain model with persistence lengths in the range of ~0.2–0.5 nm. The noise level of the force–extension curves was ~10 pN. Only the peaks with rupture forces higher than three times the noise level were included in the force histogram. The contour length increment was determined by fitting the consecutive peaks using the WLC model with fitting residuals < 20 pN. In the cases that the traces only show a spacer instead of a detectable peak, we assigned them as the signature of the unfolded protein domain because the unfolding of the protein structure is the prerequisite for the hydrolysis of the ester bond.

### 3.6. Immunofluorescence of Human Mesenchymal Stem Cells

For the immunostaining analysis, HMSCs were cultured in cell culture plates for 48 h. Then, the cells were fixed in 4% (*v*/*v*) paraformaldehyde for 20 min and treated with 0.1% Triton X-100 for 10 min. After blocking with 0.2 mM BSA, linear PEG, or Y-shape PEG for 45 min to minimize nonspecific binding separately, 4′,6-diamidino-2-phenylindole (DAPI) and phalloidin diluted with dilution buffer were added to the fixed cells and incubated overnight at 4 °C. Blocking with PBS for 45 min was used as a control. Then, the primary antibody solution was decanted, and the sample was immediately washed with PBS 3 times for 3 min each time. Immunofluorescence images were obtained using a laser confocal fluorescence microscope (Olympus FV3000, Japan).

## 4. Conclusions

In summary, we reported a new method to block nonspecific interactions in molecular and cellular biology using grafted hydrophilic polymers. Due to the special structure close to the brush shape [51,52,53,54], the effect of shielding the nonspecific interaction on the surface of the object is greatly improved. With Y-mPEG as the model molecule, we verified the effects of nonspecific interaction blocking in SMFS experiments based on AFM and fluorescence staining of hMSCs. By modifying substrates and cantilevers with Y-mPEG, the nonspecific interaction signals were significantly weakened, while the sample rate of single molecule events was enhanced in SMFS. Furthermore, the concept of preventing nonspecific interactions using Y-mPEG was extended to the fluorescence staining of cells. Y-mPEG exhibited better performance than BSA and linear PEG in blocking nonspecific interactions in the fluorescence staining of hMSCs without reducing the specific binding of fluorescent molecules. It will be our follow-up pursuit to study the industrial application of shielding nonspecific interactions utilizing topologically specific PEG.

## Data Availability

All data are available in the main text or the Supplementary Materials.

## Supplementary Materials

The following supporting information can be downloaded at: https://www.mdpi.com/article/10.3390/ijms241512414/s1.
