# Supplementary material for: Blocking Nonspecific Interactions Using Y-Shape Poly(ethylene glycol)
Source: Int J Mol Sci. 2023 Aug 4;24(15):12414. doi: 10.3390/ijms241512414 (PMC10419274; doi:10.3390/ijms241512414)
Supplement: Supplementary file 1 [file ijms-24-12414-s001.zip › ijms-2526985-supplementary.pdf]

Supplementary Information for

Blocking nonspecific interactions using Y-shape poly(ethylene glycol)

Methods

Raman spectrum

In a typical measurement, SiO_2 substrates were characterized with a confocal Raman microscope (alpha300, WITec) with a 532 nm laser under atmospheric conditions. The microscope was equipped with a piezo scanner (P-500, Physik Instrumente) and a 100× objective (Nikon, NA 0.6). The laser powers were less than 2 mW during the measurements. The Raman scattered light was detected by a thermoelectrically cooled CCD detector (DU401A-BV, Andor) with an integration time of 10 s and 3 accumulations. The measurement and subsequent data analysis were performed with the software ScanCtrlSpectroscopyPlus (Version 1.38, WITec) and Project FOUR (Version 4.1 WITec). The SiO_2 substrates were cut into slides (1 × 1 cm^2) and soaked in freshly prepared chromic acid overnight. After being washed with deionized (DI) water, ethanol, and acetone successively, the substrates were dried under a stream of nitrogen to produce surfaces with exposed hydroxyl groups. These substrates were immersed in an anhydrous toluene solution containing 5% (v/v) APTES (Merck, USA) at room temperature (R.T.) for 1 h. Then, they were washed with toluene and ethanol, dried under a nitrogen flow and incubated at 80 °C for 30 min. These substrates were immersed in DMSO containing 0.2 mM NHS-PEG-Mal, 0.2 mM NHS-linear PEG (mPEG) and 0.2 mM Y-shape PEG-SC (Y-mPEG) separately. Finally, these substrates were dried under a stream of nitrogen and stored dry.

Protein sequence

6×HIS-$(GB1)_4$-Cys

MSGSHHHHHHGSMDTYKLILNGKTLKGETTTEAVDAATAEKVFKQYANDNGVDGEWT
YDDATKTFTVTERSMDTYKLILNGKTLKGETTTEAVDAATAEKVFKQYANDNGVDGEW
TYDDATKTFTVTERSMDTYKLILNGKTLKGETTTEAVDAATAEKVFKQYANDNGVDGE
WTYDDATKTFTVTERSMDTYKLILNGKTLKGETTTEAVDAATAEKVFKQYANDNGVDG
EWTYDDATKTFTVTERSC

Supplementary Figures

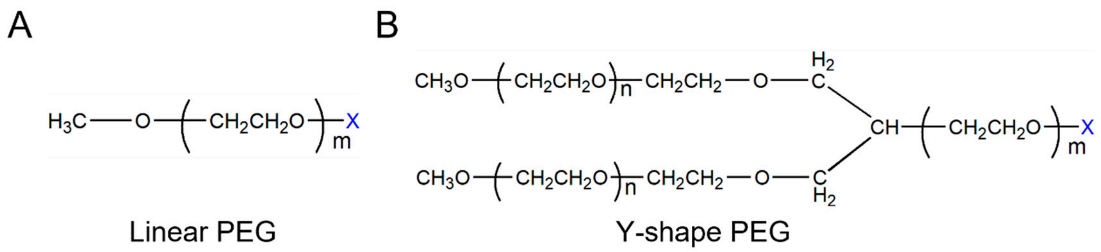

Figure S1. Chemical structures of linear PEG (A) and Y-shape PEG (B). X can be changed into various functional groups.

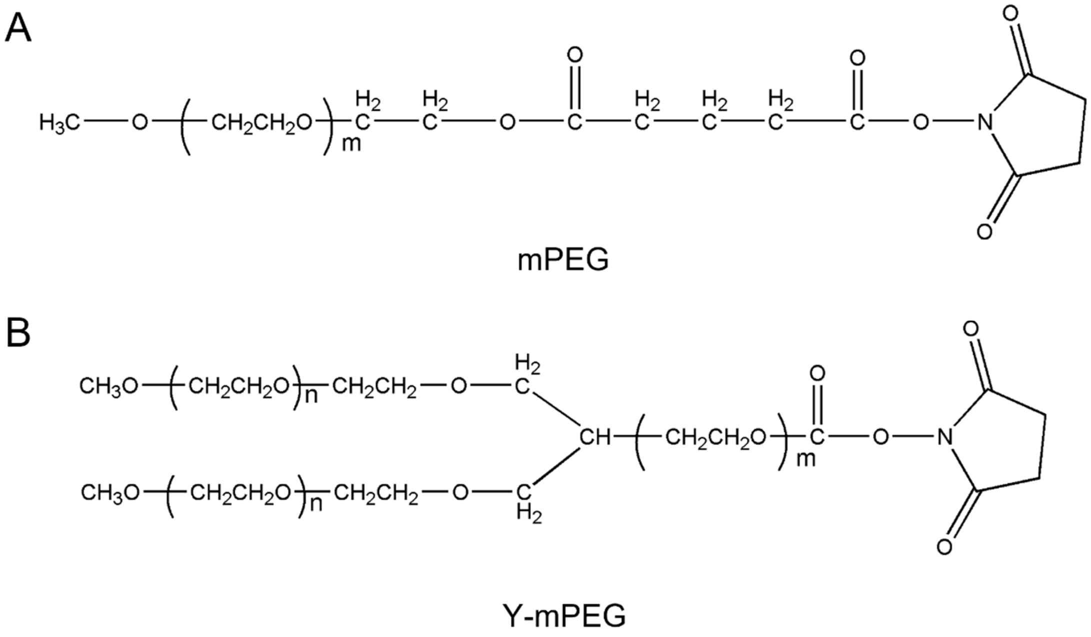

Figure S2. Chemical structures of mPEG (A) and Y-mPEG (B).

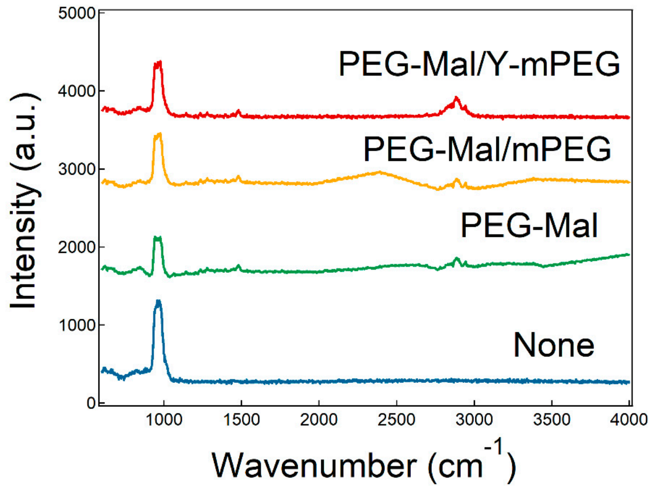

Figure S3. Raman spectrum of SiO_2 substrates modified with different PEGs. The small absorption peaks at 2883 cm^{-1} can be attributed to the contribution of low-frequency C-H vibrations from PEG. In contrast, no significant absorption peak was observed in the substrate without modification.

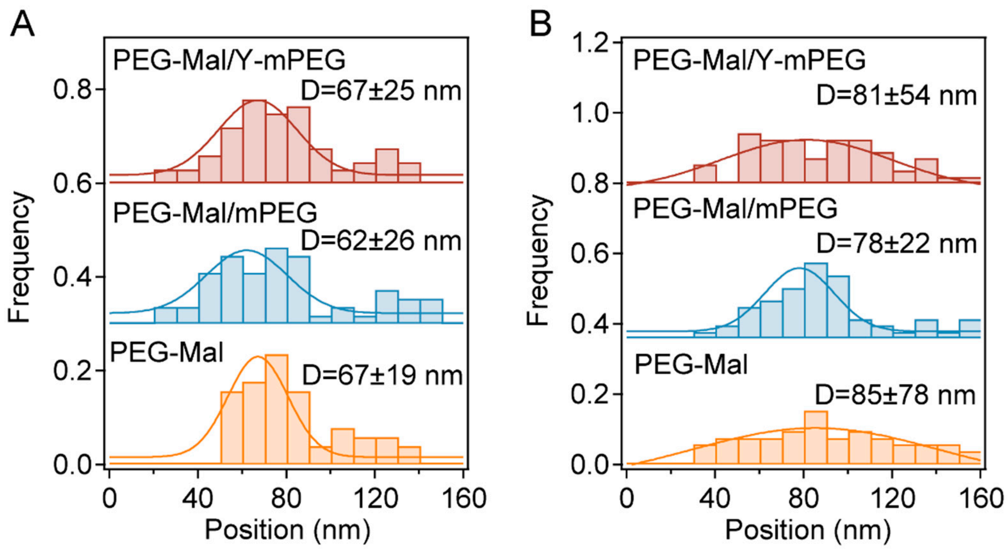

Figure S4. Position determination of the first GB1 unfolding peak. (A) Position of the first GB1 unfolding peak determined from the retract force-distance curves using different PEG-modified cantilevers. The Gaussian fitting shows average rupture positions of 67 ± 25 nm, 62 ± 26 nm and 67 ± 19 nm, respectively. (B) Position of the first GB1 unfolding peak determined from the retract force-distance curves using different PEG-modified substrates. The Gaussian fitting shows average positions of 81 ± 54 nm, 78 ± 22 nm and 85 ± 78 nm, respectively.

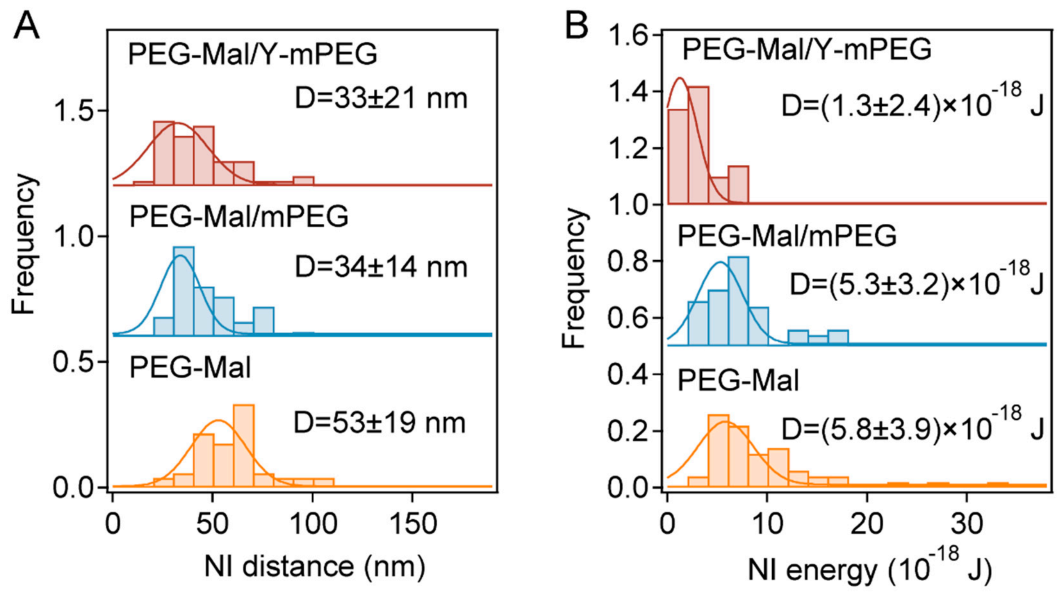

Figure S5. Histograms of fracture length (A) and energy (B) of the nonspecific interactions calculated from the retract force-distance curves in SMFS experiments using modified cantilevers (n=100). The energy of the nonspecific interactions was obtained by calculating the area of nonspecific interaction peaks. The average fracture lengths were ~33 nm, 34 nm and 53 nm in the PEG-Mal/Y-mPEG, PEG-Mal/mPEG and PEG-Mal groups, respectively. The average nonspecific interaction energies were ~1.3 $\times 10^{-18}$ J, 5.3 $\times 10^{-18}$ J and 5.8 $\times 10^{-18}$ J in the PEG-Mal/Y-mPEG, PEG-Mal/mPEG and PEG-Mal groups, respectively.

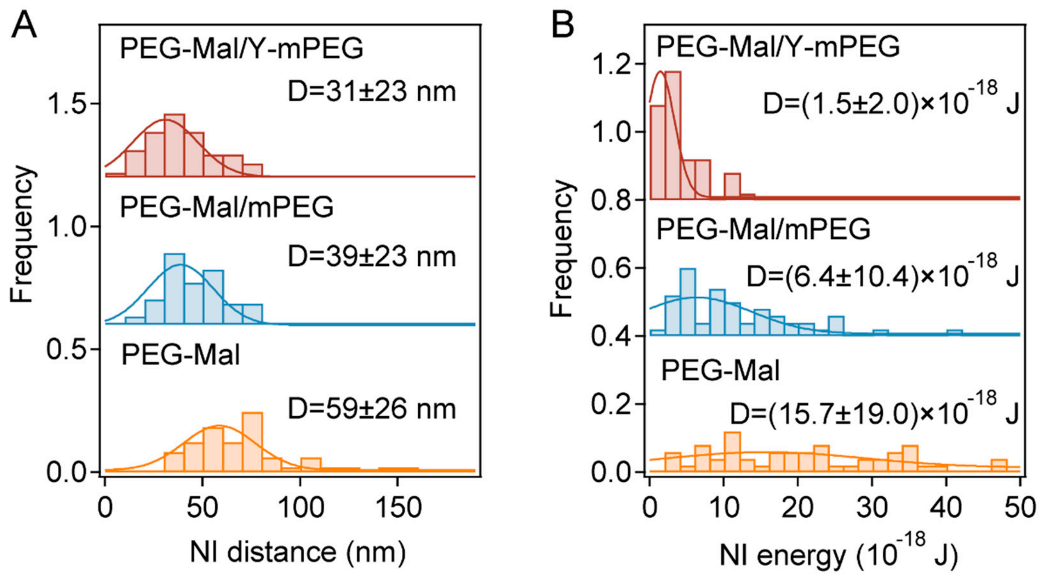

Figure S6. Histograms of fracture length (A) and energy (B) of the nonspecific interactions calculated from the retract force-distance curves in SMFS experiments using modified substrates (n=100). The energy of the nonspecific interactions was

obtained by calculating the area of nonspecific interaction peaks. The average fracture lengths were ~31 nm, 39 nm and 59 nm in the PEG-Mal/Y-mPEG, PEG-Mal/mPEG and PEG-Mal groups, respectively. The average nonspecific interaction energies were ~1.5 $\times 10^{-18}$ J, 6.4 $\times 10^{-18}$ J and 15.7 $\times 10^{-18}$ J in the PEG-Mal/Y-mPEG, PEG-Mal/mPEG and PEG-Mal groups, respectively.

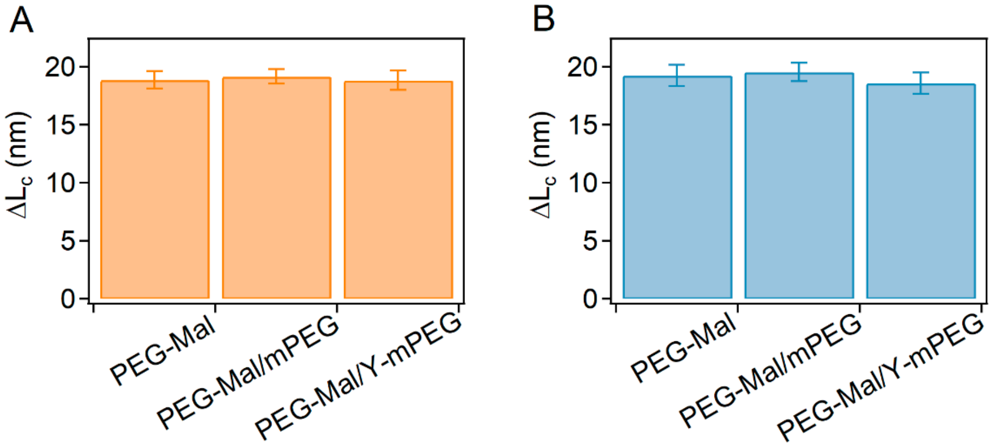

Figure S7. ΔL_c of (GB1)4-Cys when SMFS was measured using modified cantilevers (A) or modified substrates (B). ΔL_c corresponds to the distance between each protein unfolding peak.
